# Developing a sexual and reproductive health educational intervention for adolescent Syrian refugee girls: Challenges and lessons learned

**DOI:** 10.3389/frph.2022.780157

**Published:** 2022-10-03

**Authors:** Sasha Abdallah Fahme, Beatrice Khater, Myriam Dagher, Jocelyn DeJong, Sawsan Abdulrahim

**Affiliations:** ^1^ Department of Health Promotion and Community Health, Faculty of Health Sciences, American University of Beirut, Beirut, Lebanon; ^2^ Department of Family Medicine, Faculty of Medicine, American University of Beirut, Beirut, Lebanon; ^3^ Department of Epidemiology and Population Health, Faculty of Health Sciences, American University of Beirut, Beirut, Lebanon

**Keywords:** sexual and reproductive health (SRH), adolescent health, refugee health, sexual and reproductive health education, conflict and health

## Abstract

In Lebanon, a country with the highest per capita refugee population in the world, roughly one in four persons is forcibly displaced. Early marriage is highly prevalent among Syrian refugees in Lebanon and qualitative studies suggest an unmet need for sexual and reproductive health (SRH) information and services in this community. Adolescent Syrian refugee girls in Lebanon are a vulnerable population at risk of negative SRH outcomes related to early sexual debut, which occurs primarily in the context of early marriage. Despite this need, cultural norms and gender roles generally restrict adolescent girls' access to SRH resources. To address this need for comprehensive sexuality education, our team developed a novel, rights-based, peer-led, adolescent SRH educational curriculum that is specific to the context of Syrian displacement in Lebanon. This curriculum was developed to be administered as part of Project Amenah, a community-based, multi-component intervention that aims to reduce early marriage and improve SRH among adolescent Syrian refugee girls displaced in Lebanon. The curriculum, which features eight discreet age-appropriate units, is based on extensive formative work conducted in this community, as well as adaptations of early marriage programs implemented in low-resource settings elsewhere. Topics covered include, but are not limited to, gender and human rights, communication, negotiation and decision-making, reproductive anatomy, puberty and menstruation, sexually transmitted infections, family planning and modern contraception, and adolescent pregnancy. We encountered several challenges when developing this curriculum, including those related to community acceptability, varying levels of literacy levels among participants, and limited engagement with married adolescents, who may experience mobility restrictions that preclude their participation. We recommend that investigators developing adolescent SRH interventions in similar settings utilize a behavior-determinant-intervention logic model to guide their study design, elucidate community priorities and capacity by conducting preliminary qualitative work and assembling a community advisory board, and follow a peer-led model, which has shown to be effective for adolescent SRH interventions.

## Introduction

Comprehensive sexuality education has been shown to improve sexual and reproductive health (SRH) knowledge and promote safer sexual practices among adolescents in diverse low- and middle-income countries (LMICs) (1–3). Indeed, the 2018 World Health Organization (WHO) Recommendations on Adolescent Sexual and Reproductive Health strongly advocate for universal SRH education, citing the favorable impact on adolescent SRH knowledge, attitudes and preparedness (4). SRH education may be particularly important for conflict-affected adolescents in contexts of forced displacement characterized by poor accessibility to health services (5–7). Yet, there is minimal data on how SRH educational curricula may be designed and effectively implemented to address the unique needs of adolescents in conservative and patriarchal settings of displacement.

Though several SRH initiatives have been implemented in the Middle East and North Africa (MENA) region (8–11), there are few peer-reviewed evaluations of such programs, thereby restricting our understanding of their impact on communities (12). Yet, the limited available data suggest that such interventions are both acceptable and effective at improving SRH knowledge and attitudes among adolescents in MENA. For instance, an intensive school-based HIV prevention intervention among 1,964 Yemeni high school students was shown to improve HIV/AIDS knowledge scores (*p* < 0.05) and reduce HIV-related stigma (*p* < 0.01) in this population (13). Similarly, several HIV educational interventions targeting female high school students in Iran have demonstrated significant improvement in knowledge, attitudes, and behaviors (14, 15). Interestingly, a peer-led intervention in this context was shown to be superior to a traditional teacher-led model at improving HIV/AIDS knowledge (*p* < 0.001) (14). While such results are promising, there remains a large gap in SRH interventions that are not HIV/AIDS-focused and in humanitarian settings (16), including in MENA, a region characterized by protracted conflict and massive forced displacement.

There is a high need for SRH education among adolescent Syrian refugee girls in Lebanon (7, 17). While schools have been shown to be an ideal setting for SRH education (1, 2), in Lebanon, school-based SRH curricula are largely insufficient and oftentimes not delivered altogether (1, 7). Additionally, Syrian refugee children and adolescents enrolled in public schools in Lebanon typically attend afternoon sessions and receive a condensed curriculum that may not address SRH information (18). Extremely low school enrollment rates past elementary grade levels, even prior to the Covid-19 pandemic when under 10% of 15–18-year-old Syrian adolescents in Lebanon attended school (19, 20), may further preclude them from accessing such material. Notably, early marriage may be a major driver of school dropout among girls, as 93% of engaged or married adolescent Syrian refugee girls in Lebanon were found in one study to no longer attend school (21), further underscoring the urgency for delivering SRH information and services in this population, even before age 15 when the risk of early marriage starts to increase.

To address this need for SRH education, we developed a novel, rights-based, peer-led, adolescent SRH educational curriculum that is specific to the context of Syrian displacement in Lebanon. This curriculum is being administered through the Amenah Project, a community-based, multi-component intervention that aims to reduce early marriage and improve SRH among adolescent Syrian refugee girls displaced in Lebanon (22). The curriculum is based on extensive formative research conducted in this community during the Amenah pilot and the preparation phase for the actual intervention (7, 17), as well as adaptations of programs implemented in low-resource settings. To our knowledge, the Amenah Project is the first intervention offering a data-driven, contextualized approach to adolescent SRH education in this post-conflict setting of forced displacement. This paper provides a narrative reflection of the iterative processes and implementation challenges encountered when developing this SRH educational curriculum.

## Context

In Lebanon, a country with the highest per capita refugee population in the world, roughly one in four persons is forcibly displaced (23). Approximately 90% of Syrian refugees in Lebanon, of whom over half are female (24), live in extreme poverty (25). Adolescent Syrian refugee girls in Lebanon are an especially vulnerable population at risk of negative SRH outcomes related primarily to early marriage (26–30). Globally, early marriage is associated with intimate partner violence and sexually transmitted infections (STIs) including HIV infection (31–33). Further, this practice disproportionately affects girls (34), who are more likely to drop out of school and are at risk of health complications related to adolescent pregnancy (26, 35–37).

Evidence from a number of sources has shown that early marriage is high among adolescent Syrian refugees in Lebanon and qualitative studies suggest an unmet need for SRH information and services in this community (7, 17). An estimated 35% of 20–24-year-old Syrian women in Lebanon married prior to reaching 18 years of age, a figure that is higher as compared with pre-conflict Syria, though nationally representative data are lacking (28, 29, 35, 38). Pregnancy is common among married Syrian girls, with one study demonstrating that 61% of married adolescent girls younger than 18 had experienced at least one pregnancy, while over two thirds of married Syrian refugee women ages 18–24 reported not using any form of contraception (39). Despite this need, cultural norms and gender roles generally restrict adolescent girls' accessibility to SRH information (7, 17). These norms extend even to parent-child communication, with mothers in multiple settings throughout the MENA region expressing that cultural taboos and their own insufficient knowledge of these topics discourage them from participating in open discourse on SRH matters with their daughters (7, 40–42).

## Details of intervention

**Figure 1:**
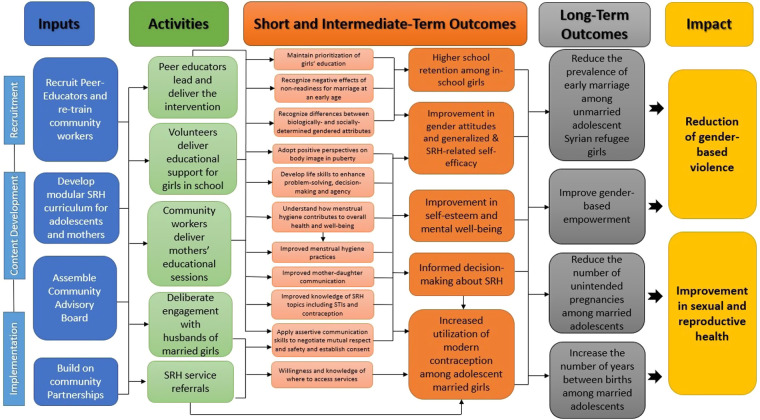
Logic model. The logic model delineates short-, intermediate- and long-term objectives of the intervention with corresponding activities and resources.

There were multiple stages involved in the development of the Amenah study curriculum, which includes SRH-specific modules. Initially, a logic model (Figure 1) was created, delineating the long-, intermediate-, and short-term outcomes of the planned intervention, from which various activities were constructed. The proposed objectives were determined in part by the research team's experiences during the Amenah pilot intervention, conducted in 2018, in which 210 Syrian refugee schoolgirls received a life-skills and general health curriculum in structured sessions (22). These sessions were facilitated by adult female Syrian community workers who reported during informal process evaluation meetings that adolescent girls lacked knowledge of even basic SRH topics such as menstruation and puberty, thus prompting a more explicit focus on SRH in the larger-scale intervention.

To better understand the health needs of this population, as well as how, where, and from whom adolescent Syrian refugee girls access SRH information and services, we conducted in-depth interviews and focus group discussions with girls, their mothers, and community stakeholders working in the health and education sectors. Published elsewhere (7, 17), the studies revealed that girls in this community have limited SRH knowledge before menarche and marriage, as well as poor access to health services despite having a perceived high burden of reproductive tract infections, unintended pregnancy, and health issues related to sexual violence. These findings influenced our SRH curriculum in several ways. For instance, we developed a dedicated curriculum for spouses of adolescent girls and expanded upon a pre-existing SRH communication curriculum for mothers, as both groups were identified in our research as primary decision-makers for adolescent girls in this population (17). Our qualitative work also showed that, despite high engagement in antenatal care, girls were rarely retained in postnatal care, which can be an excellent setting for family planning counseling and initiation of contraception (17). As such, we included a dedicated session on the potential sequelae of labor and delivery and the importance of postnatal care on maternal and neonatal health.

The curricular content was informed too by a literature review of SRH interventions conducted in Arab and Muslim-majority countries, which yielded 34 peer-reviewed studies. An additional six SRH educational curricula were identified in the grey literature, including two of which had been developed in Lebanon and Syria (11, 43). We adopted aspects of a number of the interventions identified in the review into our program. These include a dedicated curriculum on SRH communication for mothers of adolescent girls, and a peer-led education model in which university-age Syrian refugee women facilitate sessions with girls (11, 14, 44). Additionally, with the goal of increasing family planning uptake, we incorporated messages from the Qur'an supporting contraception use and developed targeted content to engage young men in the community (45, 46). The lead investigators of several of these studies participated in a workshop held by our study team in which they shared insights and recommendations for adapting their initiatives to the context of Syrian displacement in Lebanon.

**Figure 2:**
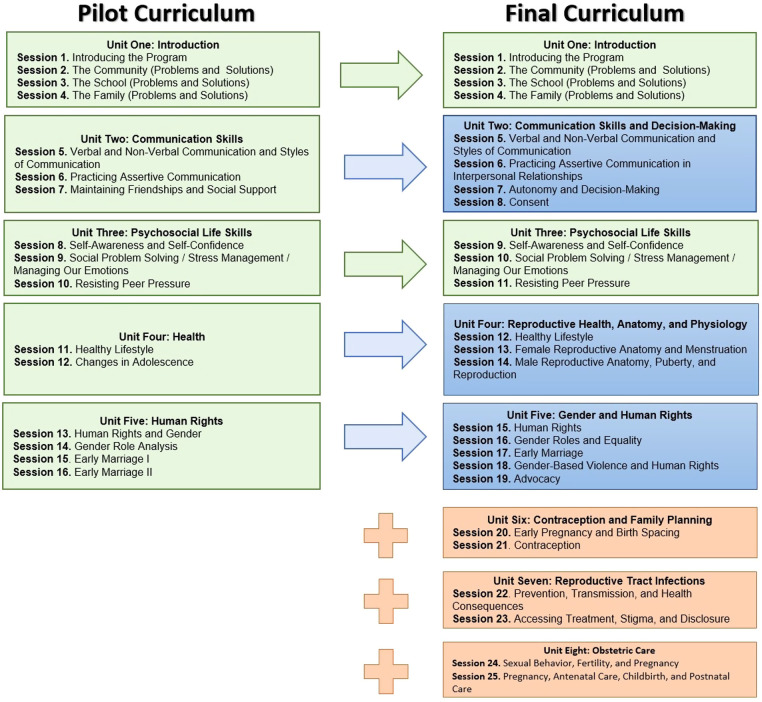
Evolution of the Amenah Project curriculum. Content in green is unchanged from the pilot study. Content in blue is based on the pilot study but significantly expanded. Content in orange is new sexual and reproductive health material.

The final curriculum includes eight age-appropriate units. As illustrated in Figure 2, five out of the eight units were derived from the pilot intervention, including content on assertive communication, gender roles, and early marriage. Other topics expanded substantially on information presented in the pilot and include autonomy and decision-making, consent, SRH rights, and male and female reproductive anatomy. In addition, three SRH-specific units were introduced for older and/or married girls, and include material on contraception, family planning, STIs, and adolescent pregnancy, among others. These specific topics were felt to be both contextually significant and culturally acceptable, and were selected based on expert opinion, similar interventions in other settings, and community feedback. The curriculum encourages participants to adopt a critical analysis of the structural and sociocultural determinants of early marriage in order to understand how these factors directly impact their own lives. The curriculum was designed to be dynamic, presenting information in an interactive rather than didactic manner, through story-telling, role-play, and arts and crafts, among others.

Project Amenah was planned for implementation in Spring 2020, after we had recruited and conducted baseline data collection on 429 adolescent Syrian refugee girls displaced in the Beqaa governorate of Lebanon. However, the intervention was delayed until Fall 2021 due to the Covid-19 pandemic and multiple economic and political humanitarian crises in Lebanon, which conferred additional, pragmatic challenges. The multi-pronged intervention features two components. The primary component is the peer-led, rights-based SRH educational intervention with adolescent refugee girls, which is the focus of the remainder of the paper. The second engages the girls' mothers and focuses on SRH communication. This latter component is being led by the same community workers who conducted the pilot study in 2018, as these women have developed strong relationships in this community. Both components of the intervention are currently ongoing.

## Discussion

We will focus below on how the content of this curriculum was specifically developed for the context of Syrian displacement in Lebanon, the challenges we encountered, and recommendations for investigators who similarly aim to adapt SRH educational curricula within resource-limited and conservative settings. Table 1 includes a summary of these recommendations and the supporting evidence, when available.

**Table 1:** Recommendations with supporting evidence/examples for the development and implementation of an adolescent SRH intervention in a LMIC setting.

Recommendation	Supporting Evidence and/or Examples
Construct a behavior-determinant-intervention (BDI) logic model to develop SRH activities intended to fulfill outcomes of interest.	BDI logic models illustrate the causal mechanisms of evidence-driven intervention activities on health behavior and outcomes and facilitate intervention evaluation (47).
Conduct formative research in the community to understand health needs and priorities.	Formative qualitative research in this community demonstrated that adolescent girls and their mothers in this community have a high and unmet need for SRH information and resources (7, 17).
Pilot test intervention to ensure that the content and model are feasible.	Unpublished data from the Amenah pilot study demonstrated that Syrian refugee girls have generally low knowledge of SRH and that discussing issues related to puberty and menstruation is generally acceptable in this community.
Ensure that the language is both direct and accessible to an adolescent population.	Prior studies exploring language preferences for text-message-based adolescent interventions found similar results (48, 49).
Include participants irrespective of schooling status and provide content in a manner appropriate to their education and literacy level.	The 2018 WHO Recommendations on Adolescent Sexual and Reproductive Health and Rights advise including out-of-school adolescents in SRH educational initiatives as this population is at greatest risk of poor SRH outcomes and lacks access to school-based SRH informational programs (4).
Recruit peer educators from the community who can both inform the SRH content and relay educational materials to participants.	Peer-led SRH educational programs in diverse Arab- and Muslim-majority countries have been determined to improve adolescent SRH knowledge and attitudes (13, 14, 44, 50–53).
Assemble a community advisory board to provide feedback on community priorities and acceptability, and facilitate messaging.	Community advisory boards with relevant stakeholders including religious leaders have been found in diverse LMICs to help facilitate the design and delivery of SRH programs (54, 55).
Age-appropriate SRH educational programs should be administered to young girls prior to marriage.	Though sociocultural norms may limit unmarried girls’ access to SRH information, age-appropriate SRH programs should be administered to girls prior to marriage, as girls often experience considerably less mobility following marriage and therefore may have minimal access to SRH information and services (56, 57).

### Translation to arabic

The curriculum includes both original content as well as material adapted from other interventions such as those developed by the Population Council and Save the Children in multiple LMICs including Bangladesh, Egypt, Ethiopia and Lebanon (8–11, 58, 59). Activities from other curricula were modified to be more applicable to the Syrian context. For instance, one exercise from a U.S.-based program that demonstrated consent by posing a hypothetical discussion between friends regarding pizza topping preferences was revised slightly to include a conversation about “man’ouche”, a popular breakfast sandwich common in Lebanon and Syria (60).

All of the material developed beyond what was included in the pilot curriculum was initially written in English. This process was led by an internal medicine physician with clinical experience caring for Syrian refugee women and adolescents, and overseen by an adolescent health specialist. The curriculum then was translated to Arabic by two translators and underwent multiple revisions by other members of the study team for completeness and clarity of the Arabic content.

Several questions arose during this process related to structural differences between formal and spoken Arabic, the latter of which varies both within and among Arab countries. Though educational textbooks are written in formal Arabic, our team felt that this more rigid and potentially unfamiliar language may be confusing and even isolating for some girls who dropped out of school at an early grade. Particularly given that the curriculum focuses not only on biomedical aspects of SRH, but also personal, and possibly even traumatic aspects of SRH rights, including sexual violence, consent, and intimate partner communication, we felt it was essential that the language itself be familiar and accessible, and efforts were made to use simple standard Arabic when possible. However, when translating certain terms, for instance, those describing male and female reproductive organs, we elected to maintain formal Arabic, which may be more appropriate and familiar to participants who attend religious classes. The suitability of the language was further assessed by advisory board members, university colleagues, and community workers (see below).

### Recruitment and training of peer educators

To deliver the curriculum content through interactive sessions, we initially recruited and trained 23 female and two male peer educators, all but two of whom were young Syrian adults living in the community.^1^ Following extensive implementation delays, we retained eight peer educations and trained an additional two. We additionally retained five female community workers who had implemented the pilot intervention to both mentor and coach the peer educators and lead the mothers' component of the intervention. Following an intensive, three-day training by an expert trainer, ongoing content trainings with the peer educators and community workers are being held at regular intervals, during which time they are given an opportunity provide feedback on their experiences facilitating sessions with study participants, resulting in real-time modifications to the curriculum, further grounding the intervention into the context of Syrian displacement in Lebanon.

Several critical issues have been raised during these training sessions. Initially, peer educators expressed reservations about discussing certain topics with adolescent girls, such as sexual pleasure, and requested removing references to the function of the clitoris, which achieved consensus among the study team. However, while the peer educators voiced similar concerns raised around other potentially stigmatizing subjects, such as STIs and contraception, these were kept in the curriculum in their entirety. The decision to retain these topics was influenced largely by participants' parents, who deemed them to be acceptable, potentially reflecting the normalization of SRH communication, a specific objective of the intervention with participants' mothers. Peer educators additionally made suggestions on how to make the sessions more interactive, through the inclusion of competitive games and group activities, which were added to the curriculum. Peer educators made further modifications to encourage collective problem-solving in the larger group, as they found that many participants had experienced traumatic events, which they were hesitant to share. Finally, feedback from peer educators alerted the study team to the importance of tailoring the curricular content on menstrual hygiene to address the lack of water, sanitation, and hygiene infrastructure in tented settlements where many refugees live, offering creative suggestions on how to maintain hygiene with minimal resources.

### Community advisory board

We assembled a Community Advisory Board (CAB) representing a diverse range of community expertise. The eight board members consist of an early married divorced Syrian refugee adolescent and her mother, two public-school educators who teach SRH curricula, a public-school guidance counselor, the director of a local NGO that addresses refugees' health and educational needs, an obstetrician/gynecologist, and a religious leader who presides over marriage cases in local family court. All CAB members live and/or work in the community of interest.

The purpose of the CAB was not to regulate the content included in the curriculum, but rather, to contribute insight on community priorities and provide recommendations on how the material may be presented in an accessible manner. Further, CAB members were also asked to help address challenges related to acceptability of the program and enhance community buy-in to promote sustainability. Specifically, the CAB reviewed the language of the curriculum to ensure that the terms used could be understood by girls of different age groups. They also examined the activities and discussion topics to assess if these were relatable and culturally acceptable by adolescent girls and their parents, respectively. When queries arose regarding specific topics, study personnel met with representatives individually to deliberate solutions that would preserve the comprehensiveness of the material.

There were several important issues raised by the CAB. Firstly, there was a robust discussion about drivers of early marriage in this population. While several members argued that limited educational opportunities in Lebanon may be influencing early marriage among Syrian refugee girls, others expressed their belief that early marriage, a practice which they argued long pre-dates the Syrian civil war, is primarily a product of cultural norms rather than conflict and forced migration. Other representatives reconciled that both cultural- and displacement-related factors could be influencing decisions around early marriage, and that educational retention and improved literacy may possibly be protective. Ultimately, there was broad consensus that the study team should construct clear, evidence-based, and concise messaging that explains the harmful effects of early marriage on adolescents, and refer to these points when discussing the intervention within the community. While drivers of early marriage among adolescent Syrian refugee girls may be varied (17, 61). the CAB acknowledged the negative effects of early marriage and recognized the importance of providing adolescent girls information about SRH.

Other recommendations to promote both study retention and girls' empowerment centered around adolescents' limited autonomy in this context, with several representatives suggesting deliberate and sustained engagement with mothers, reinforcing original plans by the study team, and young men in the community. There was general agreement, too, that substantial incentives, monetary or otherwise, are necessary to overcome competing financial interests which may contribute to attrition, particularly among men. These recommendations are supported by our qualitative work in this community (17), as well as SRH interventions elsewhere which showed parental inclusion in SRH educational programs to be associated with positive health outcomes among adolescents (62–64).

Finally, we received compelling feedback regarding community acceptability of the intervention. Several representatives agreed upon the importance of explicitly discussing girls' SRH rights, citing examples of sexual violence and coercion they are aware of as justification. The CAB also recommended holding multiple town-hall-style discussions with parents in which the rationale of presenting SRH content, and specifically contraception, to unmarried adolescent girls is clearly explained, in order to establish trust and proactively address potential concerns that disseminating information about SRH would lead to sexual promiscuity among girls. Interestingly, the board felt that such meetings would be most effective if led by study personnel, whom they thought could relay such information in an objective and data-driven manner, rather than by community leaders and religious figures, who reportedly may be perceived as less knowledgeable.

### Institutional review board

As the Amenah study targeted adolescent girls and refugees, it was judged to require a full board review by the university's Institutional Review Board (IRB). Further, the study's inclusion of explicit SRH content in its curriculum heightened the IRB's fear of risking the university's reputation by approving a study that community members may deem inappropriate. The study team thus received and responded to numerous IRB comments related to the cultural appropriateness of the intervention's content particularly for unmarried adolescent girls. In some cases, the team needed to combine scientific evidence and feedback from a CAB member in responding to an IRB recommendation. Following a prolonged review process, during which the study team provided evidence of the need for such interventions and support from the CAB, the intervention component of the study—including the curriculum—received IRB approval.

### Participant recruitment and baseline data collection

We deliberately expanded our study population from that of the pilot intervention to include out-of-school girls, who represent approximately one third of our sample. This decision is supported by WHO recommendations which recognize that adolescents at highest risk of adverse SRH outcomes are least likely to be enrolled in school (4). We observed significant variability not only in participants' critical analysis, but also in their reading skills, such that nearly 30% of girls surveyed had low literacy levels. To accommodate these needs and ensure that the curriculum would be accessible to girls of all educational backgrounds, we sought guidance from academicians with expertise in education. We then revised our curriculum and developed a parallel set of units in which activities were no longer reliant upon reading or writing, and provided tangible examples to help illustrate some of the more abstract concepts. The final outcome was two versions of the curriculum, the original one and an oral curriculum for low-literacy girls, presented in a colloquial Syrian dialect, complimented as well by the use of visual aids, which offers a comprehensive and holistic perspective of women's health and human rights.

Our team encountered multiple challenges during recruitment and data collection. Firstly, it was difficult to recruit married adolescents even just for survey completion, let alone participation in the intervention. Aligned with findings from the qualitative literature on Syrian refugee girls (56, 57), our experiences suggest that once girls in this community are married, they encounter comparative limitations to their mobility, making it difficult for us to recruit and retain them in our intervention. This also implies that married girls, of whom the overwhelming majority are no longer in school, have limited access to SRH information following marriage, despite their high risk of adolescent pregnancy. These observations underscore the importance of engaging with girls in this community prior to marriage, not only to improve their SRH knowledge, but also to strengthen their self-efficacy. Indeed, improvement in general and SRH-specific self-efficacy are two outcomes of interest in this study, and activities designed to promote these attributes were incorporated throughout the curriculum.

### Study evaluation

The intervention, which is currently ongoing, will be evaluated in a quasi-experimental study through a pre- and post-intervention evaluation. An interviewer-administered survey will assess a number of knowledge and attitudinal outcomes related to menstruation, early marriage, and education among all participants. Participants ages 15 and older will additionally complete a survey examining knowledge and beliefs related to contraception and STIs, including HIV infection. Married participants of all ages will be asked additional questions regarding their personal experiences with family planning and other SRH services, as it was felt to be culturally inappropriate to ask unmarried adolescents behavioral questions related to sexual activity.

## Acknowledgement

Following the development of the curriculum, we encountered several pragmatic challenges related to humanitarian crises in Lebanon that delayed the implementation of the intervention. Since Fall 2019, Lebanon has experienced protracted political and economic crises, precipitating closure of public schools, which impacted recruitment for this study (65). The financial collapse, declared by the World Bank to be among the three worst economic crises globally since the mid-19th century (66), and characterized by a roughly 90% depreciation of the Lebanese Pound (67), led to capital control measures that further impacted the study by complicating payment procedures for study personnel. Additionally, the Lebanese government lifted subsidies on essential goods, precipitating a severe fuel shortage which has substantially limited transportation within the country and thereby restricted study activities (68). These ongoing crises were substantially worsened by the Covid-19 pandemic, which prolonged school closures such that the vast majority of Syrian refugee students in Lebanon experienced an approximate two-year hiatus in schooling, with implications for early marriage and risk of child labor (69, 70). While public health measures precluded in-person delivery of the curriculum, widespread disruptions in electricity and internet limited any virtual activities as well, though study personnel have been able to communicate informally with participants by telephone. Despite these limitations, the intervention began in Fall 2021, with recruitment of 339 adolescent girls; at the time of this writing, the intervention is ongoing.

## Conclusion

In conclusion, there is an unmet need for SRH information among adolescent Syrian refugee girls displaced in Lebanon, which we aim to address through an evidence-based, peer-led SRH educational curriculum. The curriculum is based on formative work, including a pilot intervention conducted in this community, as well as interventions implemented in other settings which have been adapted to the context of Syrian displacement. Though implementation of the intervention has been delayed due to ongoing humanitarian crises, we were able to successfully recruit 339 participants. Investigators conducting SRH interventions among similar populations of displaced adolescents may benefit from investing time and resources to understand the specific priorities and capacity of the community which they intend to serve. Pilot-testing the curriculum and recruiting community members to both inform the content and facilitate delivery may promote efficacy and sustainability of any SRH intervention.

## Data Availability

The original contributions presented in the study are included in the article/Supplementary Material, further inquiries can be directed to the corresponding author/s.

## Footnotes

^1^One peer educator was Palestinian and one was Lebanese but both were also living in the community of interest.
